# Comparisons of Metabolic Load between Adaptive Support Ventilation and Pressure Support Ventilation in Mechanically Ventilated ICU Patients

**DOI:** 10.1155/2020/2092879

**Published:** 2020-01-28

**Authors:** Yen-Huey Chen, Hsiu-Feng Hsiao, Hui-Wen Hsu, Hsiu-Ying Cho, Chung-Chi Huang

**Affiliations:** ^1^Department of Respiratory Therapy, College of Medicine, Chang Gung University, Taoyuan, Taiwan; ^2^Department of Pulmonary and Critical Care Medicine, Chang Gung Memorial Hospital, Linko, Taiwan; ^3^Department of Respiratory Care, Chiayi Campus, Chang Gung University of Science and Technology, Chiayi, Taiwan; ^4^Department of Respiratory Therapy, Chang Gung Memorial Hospital, Linko, Taiwan; ^5^Department of Thoracic Medicine, Fu Jen Catholic University Hospital, New Taipei City, Taiwan

## Abstract

**Purpose:**

The aim of this study was to compare the metabolic load between adaptive support ventilation (ASV) and pressure support ventilation (PSV) modes in critically ill patients.

**Methods:**

Sequential 20 min ventilation by PSV followed by 20 min ASV in critically ill patients was assessed. ASV was set for full support, i.e., with the minute volume control set at the same level as the minute volume observed during PSV. The trial started from PSV 8 cmH_2_O and continued with high (PSV 12 cmH_2_O) to low (PSV 0) conditions or low to high conditions, in random order. The oxygen consumption (VO_2_), production of carbon dioxide (VCO_2_), and energy expenditure (EE) were measured by indirect calorimetry (IC).

**Results:**

Twenty-four patients with critical illness participated in the study. Comparing with the PSV mode, the EE in the ASV mode was lower in the level of PSV 0 cmH_2_O (1069 ± 73 vs. 1425 ± 76 kcal), PS 8 cmH_2_O (1116 ± 70 vs. 1284 ± 61 kcal), and PS 12 cmH_2_O (1017 ± 70 vs. 1169 ± 58 kcal) (*p* < 0.05). The VO_2_, VCO_2_, and P0.1 in PSV were significantly higher than those in ASV (*p* < 0.05). The VO_2_, VCO_2_, and P0.1 in PSV were significantly higher than those in ASV (

**Conclusion:**

In patients with critical illness, the application of ASV set for full support was associated with a lower metabolic load and respiratory drive than in any of the studied PSV conditions.

## 1. Introduction

For patients with acute respiratory failure, mechanical ventilation (MV) is the cornerstone of management. Patients admitted to the intensive care unit (ICU) who require MV are expected to have higher mortality rates than those who do not require respiratory support [1]. The application of the mechanical ventilator is crucial for patients with critical illness, especially during the weaning process. Choosing a ventilator mode that maintains adequate oxygenation and ventilation without inducing pulmonary complication or delaying the weaning process is a critical concern for clinicians.

Pressure support ventilation (PSV) is a widely used mode of ventilation, especially during the weaning process for critically ill patients [2]. During ventilation, PSV provides a fixed end-inspiratory pressure, regardless of the variabilities of patient's demand for ventilator or gas exchange [3–6]. Thus, the preset values for ventilator parameters, such as a fixed end-inspiratory pressure, are unlikely to provide appropriate assist immediately.

Adaptive support ventilation (ASV) is one of the recently developed MV modes. It is a closed-loop pressure-controlled ventilation mode. ASV is a mode of ventilation which allows delivery of pressure cycles that may be assisted (PSV-like) or controlled (PC-like). The characteristics of the cycles delivered by the ventilator are related to a minute ventilation target set by the clinician and automated measurements of the patient's respiratory mechanics [7]. The working principles of this mode are based on the findings of Otis et al., suggesting that for a given level of alveolar ventilation and dead space, there is an optimum combination of respiratory rate and tidal volume delivered in terms of the respiratory work of breathing (WOB) [8]. In this context, “Optimal” means minimizing mechanical WOB. During this mode, any changes in respiratory mechanics and effort of the patient are accompanied by a dynamic pattern of breathing that gradually guides the patient to the new goal where optimal breath is achieved. Therefore, maybe more energy efficient to minimize the cumulative effects of elastic and resistive load is imposed on the respiratory system [4].

Measuring oxygen consumption (VO_2_) and carbon dioxide production (VCO_2_) can be useful for assessing the function of alveolar ventilation, pulmonary perfusion, and ventilation optimization [9]. In patients supported with MV, monitoring VO_2_ predicts the success or failure of weaning trials [10]. In addition, the VO_2_ of respiratory muscles is computed as the difference between VO_2_ measured during MV and spontaneous breathing. This approach assumes that the VO_2_ of respiratory muscles varied with and without ventilatory support [11, 12]. In addition, VCO_2_ and VO_2_ in mechanically ventilated patients can be measured using an indirect calorimeter [13]. A study reported significantly increased WOB in terms of energy expenditure (EE) and VO_2_ during T-piece breathing compared with those in the PSV mode in critically ill patients [14].

In ASV applied in passive patients, the optimal combination of Vt and RR should minimize the energy transferred by the ventilator to the respiratory system. Whether ASV applied in actively breathing patients results in a lower metabolic load, such as VO_2_ or EE when compared with a conventional ventilation mode like pressure support ventilation (PSV), remains unclear. The purpose of this study was to compare the metabolic load measured by indirect calorimetry between ASV set for full respiratory support and PSV set at different pressure levels in stable critically ill patients.

## 2. Methods

This prospective study was performed in a 24-bed medical ICU at Chang Gung Memorial Hospital, Taiwan. The inclusion criteria for this study were as follows: (1). patients who had been mechanically ventilated for more than 24 h with PSV levels of 8 cmH_2_O, PEEP ≤8 cmH_2_O, and FiO_2_ ≤40%. (2). Medical stability (arterial blood gas pH = 7.35–7.45, PaO_2_ ≥ 60 mmHg on FiO_2_ ≤40%, and PaCO_2_ 40 ± 5 mm Hg; for patients with COPD who had been chronically hypercapnic and had well tolerated the ventilator support, a PaCO_2_ value up to 55 mmHg was acceptable, absence of signs and symptoms of infection, and hemodynamic stability). The exclusion criteria included acute lung or systemic infection, hemodynamic instability, previous or ongoing neuromuscular disease (e.g., myasthenia gravis and Guillain–Barré disease), and patients with chest tubes. The study was approved by the hospital's institutional review board. The study was performed in accordance with the Declaration of Helsinki. Written informed consent forms were obtained from subjects or their relatives prior to inclusion.

### 2.1. Study Protocol

**Figure 1:**
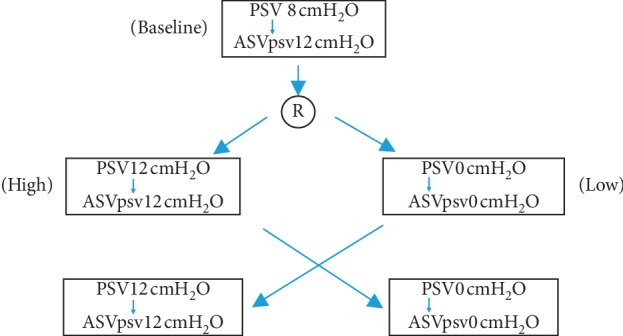
Study design. R indicates randomized.

First, the clinical characteristics were recorded, including age, sex, Acute Physiology and Chronic Health Evaluation II (APACHE II) score, and diagnosis. Patients were monitored until their discharge from the ICU or until death. All patients were ventilated with a microprocessor-controlled ventilator that had a closed-loop ventilation capability (Galileo Gold; Hamilton Medical, Rhäzüns, Switzerland). After enrollment, the bronchial hygiene therapy was performed first, followed by lying and the bed head at 45° for 5–10 minutes before starting the IC measurements. All measurements were performed at both PSV and ASV modes under 3 ventilator support levels: low (PSV 0 cmH_2_O, PSV0), baseline (PSV 8 cmH_2_O, PSV8), and high (PSV 12 cmH_2_O, PSV12). We conducted a randomized crossover controlled trial. Patients were randomized using a computer-generated randomization sequence in sealed envelopes to receive ventilator support levels in the sequences of low-to-high or high-to-low conditions. All patients started from baseline level (PSV 8). After the baseline period, patients were then randomized into groups with different sequences: low (PSV0) to high (PSV12) or high (PSV12) to low (PSV0) conditions (Figure 1). The pressure support level of PSV0 was set at zero. In each level, the ventilator mode started from PSV modes for 20 minutes. After confirming that patients were well tolerated with the minute volume of the PSV mode without signs/symptoms of respiratory distress, the ventilator mode was then switched to the ASV mode for 20 min. The minute volume of the last 5 min of each PSV level was recorded and averaged. The result was used to set the minute volume target (i.e. the %MinVol control) of the following ASV step of the study. At the beginning of each matched-ASV level, the %MinVol was adjusted until the measured minute volume (which is measured by the ventilator) of the patient was similar to that in the previous PSV mode. The rest of the ventilator parameter setting remained the same as those before enrolling the study. Besides IC parameters, the respiratory parameters such as mean airway pressure (MAP), peak pressure (Ppeak), airway occlusion pressure at 0.1 s (P0.1), spontaneous RR, Vt, and minute volume were recorded from the mechanical ventilator in the last 5 min of each condition.

### 2.2. Indirect Calorimetry (IC) Measurement

A calorimeter module (Engström Carestation, General Electric, Madison, WI) was used for indirect calorimetry. The module consists of a fast differential paramagnetic oxygen analyzer, an infrared analyzer for carbon dioxide, and a pneumotachograph to measure the inspired and expired volumes. The pneumotachograph and gas sampling ports were connected to a disposable connector, placed between the *Y*-piece of the ventilator circuit and the endotracheal tube. The signals from the pneumotachograph and gas analyzers were synchronized for breath-by-breath data on gas exchange. Under each condition, the data were collected for 30 min with the first 10 min being discarded for analysis purposes. We consider the steady state to be the point after 5 consecutive minutes measurement when VCO_2_ and VO_2_ vary by ±10%. During the last 5 min of each steady state, the following data were recorded: VCO_2_, VO_2_, EE, and respiratory quotient (RQ).

### 2.3. Statistical Analysis

The sample size was calculated according to a previous study [13], and assuming a middle effect size and an *α* error of 0.05, a sample size of 25 would have 80% power to detect the difference between the modes. Allowing for the 20% dropout rate, the sample size was increased to 30.

Analysis was conducted using SPSS v.17. The results were expressed as mean ± standard deviation. The variables between the PSV and ASV modes were compared using Student's *t* test. A repeated-measure analysis of variables was used to examine the difference among the three different levels in the PSV and ASV modes. The variables were correlated using Pearson correlation coefficient. *p* < 0.05 indicated statistical significance.

## 3. Results

### 3.1. Demographic Characteristics

From June 2017 to May 2018, 26 patients were enrolled in the study (Figure 1). During the study period, two subjects met the exclusion criteria (hemodynamic instability) due to their primary diagnosis and were excluded, leaving 24 subjects for the analysis.

The demographic and clinical characteristics of the subjects are summarized in Table 1. Most subjects were men (70.8%). The admission diagnosis was mostly respiratory system diseases (pneumonia 37.5%, COPD 8.3%, and other respiratory diseases 16.7%). The mean age was 70.6 ± 14.0 years, and the mean APACHE II score was 19.0 ± 4.0. During ICU admission, only one subject (4.1%) failed to extubate from the mechanical ventilator.

### 3.2. Comparison of Lung Mechanics and Metabolic Load between the PSV and ASV Modes

The metabolic load was measured (Table 2), and no significant difference was observed in the minute volumes between the PSV and ASV modes in the PSV0, PSV8, and PSV12 levels (*p* < 0.05). The EE in PSV0 (1425 ± 76 kcal), PSV8 (1284 ± 76 kcal), and PSV12 (1169 ± 58 kcal) was significantly higher than that in the ASV mode with matched minute volumes (1069 ± 73, 1116 ± 70, and 1017 ± 70 kcal, respectively) (*p* < 0.001). VO_2_ in PSV0 (218 ± 11 mL/min), PSV8 (195 ± 9 mL/min), and PSV12 (178 ± 8 mL/min) was significantly higher than that in the ASV mode with matched minute volumes (161 ± 10, 169 ± 10, and 154 ± 10 mL/min, respectively) (*p* < 0.001). VCO_2_ was also significantly higher in the PSV mode than that in the ASV mode in each level (*p* < 0.001).

In the measurement of lung mechanics (Table 3), a significant difference in Vt, RR, and P0.1 was observed between the PSV and ASV modes. In the PSV mode, Vt in PSV0 (442 ± 25 mL), PSV8 (498 ± 30 mL), and PSV12 (501 ± 30 mL) levels was significantly lower than that in the ASV mode (520 ± 23, 539 ± 27, 529 ± 26 mL, respectively) (*p* < 0.001). RR in the PSV mode was significantly higher than that in the ASV mode with matched minute volumes. P0.1 in PSV0 (−4.4 ± 0.4 cmH_2_O), PSV8 (−3.3 ± 0.4 cmH_2_O), and PSV12 (−2.7 ± 0.3 cmH_2_O) was significantly higher than that in the ASV mode (−2.1 ± 0.2, −2.0 ± 0.2, and −1.8 ± 0.2 cmH_2_O) (*p* < 0.001). Ppeak and MAP in PSV0 and PSV8 were significantly lower than those in the ASV mode with matched minute volumes (*p* < 0.001).

### 3.3. Comparison of Measurements among 3 PSV Levels and 3 ASV Trials

In the comparison of measurements among 3 different PSV levels (Table 4), there was a significant difference in the measurement of EE, VO_2_, and VCO_2_. The EE in PS12 (1169 ± 58 kcal) was significantly lower than that in PS8 (1284 ± 61 kcal) and PSV0 (1425 ± 76 kcal) (*p* < 0.001). In addition, the EE in PS8 was also significantly lower than that in PSV0. This indicated that a higher support level was associated with a lower metabolic load in the PSV mode. The Vt, Ppeak, and MAP were significantly higher in PS12 when compared with those in PS8 and PSV0 (*p* < 0.001). The Vt in PS12 (501 ± 30 ml) was significantly higher than that in PS8 (498 ± 30 ml) and PSV0 (442 ± 25 ml) (*p* < 0.001). The RR and P0.1 were significantly lower in PS12 than those in PS8 and PSV0 (*p* < 0.001). Among the 3 ASV trials, no significant difference was found in the %MinVol setting, as well as in the measurements of tidal volume, frequency, inspiratory pressure above PEEP, and P0.1 (Table 5). The corresponding metabolic measurements of EE, VO_2_, and VCO_2_ showed some differences, whose amounts were clinically irrelevant although statistically significant (Table 5).

### 3.4. Correlation between Lung Mechanics and Metabolic Load in the PSV and ASV Modes

After pooling data from PSV and ASV modes, a significant correlation was observed among the mechanical load and metabolic load. P0.1 was positively correlated with EE (*r* = 0.442), VO_2_ (*r* = 0.424), and VCO_2_ (*r* = 0.457) (Table 6).

## 4. Discussion

This study compared the metabolic load of PSV and ASV. We discovered that when the %MinVol control of ASV was set to match the minute volume observed during PSV with PS from zero to 12 cmH_2_O, ASV was associated with a larger Vt and lower RR than PSV even when the latter was set at the top level of 12 cmH_2_O. This was due to the generation of PS levels by ASV larger than by PSV and was associated with lower EE, VO_2_, VCO_2_, and P0.1 during ASV.

In our study, EE and VO_2_ in the ASV mode were significantly lower than those in the PSV mode at similar minute volumes. In addition, P0.1 was lower in ASV which indicated patients maintain similar minute volumes with lower respiratory effort and respiratory muscle work in the ASV mode than those in the PSV mode. This could be part of the reasons that contribute to the lower metabolic load in the ASV mode. ASV is a complex, dual-controlled mode. After setting the target %MinVol, the ventilator calculates an optimal breath pattern and associated target values for tidal volume and respiratory rate according to the Otis equation [15]. The basic assumption of Otis equation is that the optimal breath pattern is identical to a patient would choose naturally in resting status [8]. In healthy individuals, the resting VO_2_ from the respiratory system is about only 1–2% of whole body oxygen consumption [16]. ASV monitors patients' respiratory mechanics breath by breath and automatically adjusts breathing pattern based on user inputs and the changes of respiratory mechanics. ASV ensures effective alveolar ventilation and leads patients to an optimal and comfortable breathing pattern [4]. Our result was consistent with previous studies.

P0.1 reflected both the respiratory drive and work of respiratory muscles. Our study showed that the P0.1 in the ASV mode was significantly lower than that in the PSV mode. The mechanisms for the lower metabolic work and P0.1 at similar minute volume were probably related to the higher Vt, as recommended by the ASV algorithm when ASV is set for full support, i.e., with the %MinVol control set close to the minute volume requirement of the patient. The higher Vt decreased the muscle force, and the patient has to generate to get the desired Vt. The higher Vt may also stimulate pulmonary stretch receptors, sending the impulse to central drive, to lower the respiratory rate [17–19]. This was further substantiated by the higher Vt and lower RR in ASV when compared with those in PSV in our study.

According to our inclusion criteria, only patients with stable condition (hemodynamically stable, acceptable ABG data) were recruited. This indicated that the original ventilator setting provided adequate support for patient's ventilation requirement. To reach similar VE, the %MinVol was set to an average of 144%MinVol at the presence of patients' full spontaneous breath. A similar finding has been reported in a previous study [18]. Wu et al. reported that, in patients with respiratory failure, when the % MinVol setting is increased gradually to a point where mandatory breath appeared (target point), the P0.1 was significantly decreased, compared with that in 100% MinVol [18]. The average %MinVol setting of the target point in Wu's study was 165%. Although all the measurements in our study were performed in the presence of spontaneous breath, both our and Wu's data showed that the %MinVol required to decrease WOB in patients with respiratory failure is greater than what operator manual recommended [15]. The ability of ASV to unload the patient's respiratory muscles also depends on the ASV + a given setting of (%MinVol) control of ASV. The inadequate %MinVol setting may result in increased work of breath and respiratory muscle fatigue in patients. However, an oversetting may result in highly unloading respiratory muscle and put patients under the risk of respiratory muscle atrophy. Thus, carefully adjusted setting to meet patients' requirement without inducing other complications is crucial for patients with a mechanical ventilator. The P0.1 and presence of spontaneous/mandatory breath may be the indicators when set the %MinVol. In addition, the data from IC measurements may also provide useful information to ensure appropriate ventilator setting.

The lung mechanics data reveal that Vt in PSV was significantly lower and RR was higher than those in ASV under 3 levels. In PSV, the level of pressure is fixed regardless of the ventilator demand of the patients. When the level of PSV cannot provide adequate Vt, patients must increase RR to match the demand, which may thus lead to increased WOB [20].

### 4.1. Limitations

Some limitations affected this study. First, the population was relatively small. The results reflect findings from a single institution and represent a heterogeneous collection of diseased state. However, the population reflects patients that are likely to be encountered in an ICU. Second, the data in our study were obtained by the version of ASV provided by the Galileo Gold ventilator (Hamilton Medical). The generalizability of our results to other versions requires further study. Third, we used a commercial device to measure; this system has the advantage of being noninvasive and of providing continuous measurements of VO_2_. We utilized a single device to ensure that device-device agreement was not a confounding factor in our study. However, the data may not specifically reflect the work of respiratory muscles, which may also change with the ventilatory mode. The measurements of esophageal pressure obtained through the placement of the esophageal balloon were proposed as a valid indicator of respiratory muscle work and may be considered in future studies.

## 5. Conclusion

Our study showed that, compared with PSV, ASV set for full support in actively breathing patients provides larger Vt and lower RR with lower VO_2_, VCO_2_, and EE at similar minute volume. The ventilatory drive as assessed by P0.1 was also lower during the ASV mode than during the PSV mode. Our study also shows the interest of metabolic measurements in patients with critical illness during ventilated or weaning stages.

## Figures and Tables

**Table 1:** Demographic data of participated subjects.

	(*n* = 24)	
Male/female, (*n*)	17/7	
Age (year)	70.6 ± 14.0	
APACHE II score	19.0 ± 4.1	
Days on MV (day)	9.6 ± 5.6	
ICU stay (day)	10.3 ± 5.8	
Diagnosis, *n* (%)		
Pneumonia	9	37.5%
COPD AE	2	8.3%
Other respiratory diseases	4	16.7%
Congestive heart failure	2	8.3%
Cerebral vascular accident	2	8.3%
Cancer	5	20.8%
Ventilator setting		
Pressure support level (cmH_2_O)	8	
PEEP (cmH_2_O)	8	
FiO_2_ (%)	34.5 ± 2.1	

APACHE II, Acute Physiology and Chronic Health Evaluation; COPD AE, Chronic Obstructive Pulmonary Disease Acute Exacerbation.

**Table 2:** Comparison of metabolic loads between different ventilatory modes.

	PS0	ASV _PS0_	*p*	PS8	ASV_PS8_	*p*	PS12	ASV_PS12_	*p*
MV (L/min)	9.1 ± 1	8.9 ± 0.9	0.51	9.3 ± 1.1	8.8 ± 1	0.49	8.7 ± 1	8.6 ± 1	0.51
EE (kcal)	1425 ± 76	1069 ± 73^*∗*^	<0.001	1284 ± 61	1116 ± 70^*∗*^	<0.001	1169 ± 58	1017 ± 70^*∗*^	<0.001
VO_2_ (ml)	218 ± 11	161 ± 10^*∗*^	<0.001	195 ± 9	164 ± 10^*∗*^	<0.001	178 ± 8	154 ± 10^*∗*^	<0.001
VCO_2_ (ml)	143 ± 7	116 ± 9^*∗*^	<0.001	135 ± 7	120 ± 8^*∗*^	<0.001	121 ± 7	108 ± 8^*∗*^	<0.001
RQ	0.68 ± 0.1	0.69 ± 0.1	0.902	0.67 ± 0.1	0.71 ± 0.1	0.257	0.70 ± 0.1	0.70 ± 0.1	0.899

^*∗*^ *p* < 0.05, comparisons between two modes. MV, minute ventilation; EE, energy expenditure; VCO_2_, oxygen consumption; VCO_2_, carbon dioxide production.

**Table 3:** Comparison of ventilatory parameters between different ventilatory modes.

	PS0	ASV _PS0_	*p*	PS8	ASV_PS8_	*p*	PS12	ASV_PS12_	*p*
MV (L/min)	9.1 ± 1	8.9 ± 0.9	0.51	9.3 ± 1.1	8.8 ± 1	0.49	8.7 ± 1	8.6 ± 1	0.51
Spont RR (bpm)	21 ± 1	17 ± 1^*∗*^	<0.001	19 ± 1	16 ± 1^*∗*^	<0.001	18 ± 1	16 ± 1^*∗*^	<0.001
Vt (ml)	442 ± 25	520 ± 23^*∗*^	<0.001	498 ± 30	539 ± 27^*∗*^	<0.001	501 ± 30	529 ± 26^*∗*^	<0.001
Ppeak (cmH_2_O)	12 ± 1	21 ± 4^*∗*^	<0.001	16 ± 1	21 ± 3^*∗*^	<0.001	20 ± 1	21 ± 4	0.06
MAP	9.1 ± 2	12 ± 1^*∗*^	<0.001	10.8 ± 1	12.1 ± 0.1	<0.001	11.8 ± 1	12.1 ± 1	0.07
P0.1 (cmH_2_O)	−4.4 ± 0.4	−2.1 ± 0.2	<0.001	−3.3 ± 0.4	−2.0 ± 0.2	<0.001	−2.7 ± 0.3	−1.8 ± 0.2	<0.001

^*∗*^ *p* < 0.05, comparisons between two modes. Vt, tidal volume; RR, respiratory rate; MV, minute ventilation; Ppeak, peak pressure; MAP, mean airway pressure; P0.1, airway occlusion pressure.

**Table 4:** Comparisons of metabolic and respiratory measurements among 3 PS levels.

	PS0 cmH_2_O	PS8 cmH_2_O	PS12 cmH_2_O	*p*	
EE (kcal)	1425 ± 76	1284 ± 6	1169 ± 58	<0.001^*∗*^	*c* < *a*, *b*; *b* < *c*
VO_2_ (ml/min)	218 ± 11	195 ± 9	178 ± 8	<0.001^*∗*^	*c* < *a*, *b*; *b* < *c*
VCO_2_ (ml/min)	143 ± 7	135 ± 7	121 ± 7	<0.001^*∗*^	*c* < *a*, *b*
MV (L/min)	9.1 ± 1	9.3 ± 1.1	8.7 ± 1	0.21	
Spont RR (bpm)	21 ± 1	19 ± 1	18 ± 1	0.01^*∗*^	*c* < *a*, *b*; *b* < *c*
Vt (ml)	442 ± 25	498 ± 30	501 ± 30	<0.001^*∗*^	*c* > *a*, *b*; *b* > *c*
Ppeak (cmH_2_O)	12 ± 1	16 ± 1	20 ± 1	<0.001^*∗*^	*c* > *a*, *b*; *b* > *c*
MAP (cmH_2_O)	9.1 ± 2	10.8 ± 1	11.8 ± 1	<0.001^*∗*^	*c* > *a*, *b*; *b* > *c*
P0.1 (cmH_2_O)	−4.4 ± 0.4	−3.3 ± 0.4	−2.7 ± 0.3	<0.001^*∗*^	*c* < *a*, *b*; *b* < *c*

MV, minute ventilation; EE, energy expenditure; VCO_2_, oxygen consumption; VCO_2_, carbon dioxide production; Vt, tidal volume; RR, respiratory rate; MV, minute ventilation; Ppeak, peak pressure; MAP, mean airway pressure; P0.1, airway occlusion pressure; *a*, PS0 cmH_2_O; *b*, PS8 cmH_2_O; *c*, PS12 cmH_2_O. ^*∗*^Variables comparison over 3 levels of PS were analyzed by repeated-measures ANOVA, *p* < 0.05.

**Table 5:** Comparisons of metabolic and respiratory measurements among 3 ASV trials.

	ASV_PS0_	ASV_PS8_	ASV_PS12_	*p*	
EE (kcal)	1069 ± 73	1116 ± 70	1017 ± 70	<0.001^*∗*^	*c* < *b*
VO_2_ (ml/min)	161 ± 10	169 ± 10	154 ± 10	<0.001^*∗*^	*c* < *b*
VCO_2_ (ml/min)	116 ± 9	120 ± 8	108 ± 8	0.01^*∗*^	*c* < *a*, *b*
MV (%)	145 ± 6	144 ± 6	143 ± 6	0.82	
MV (L/min)	8.9 ± 0.9	8.8 ± 1	8.6 ± 1	0.52	
Spont RR (bpm)	17 ± 1	16 ± 1	16 ± 1	0.25	
Vt (ml)	520 ± 23	539 ± 27	529 ± 26	0.14	
Ppeak (cmH_2_O)	21 ± 4	21 ± 3	21 ± 4	0.90	
MAP (cmH_2_O)	12 ± 1	12.2 ± 1	12 ± 1	0.74	
Delta P (cmH_2_O)	13 ± 4.2	13.4 ± 3.8	13.3 ± 4.3	0.94	
P0.1 (cmH_2_O)	−2.1 ± 0.2	−2.2 ± 0.2	−1.8 ± 0.2	0.12	

MV, minute ventilation; EE, energy expenditure; VCO_2_, oxygen consumption; VCO_2_, carbon dioxide production; Vt, tidal volume; RR, respiratory rate; MV, minute ventilation; Ppeak, peak pressure; MAP, mean airway pressure; P0.1, airway occlusion pressure; Delta P, difference between Ppeak and PEEP. *a*, PS0 cmH_2_O; *b*, PS8 cmH_2_O; *c*, PS12 cmH_2_O. ^*∗*^Variables comparison over 3 levels of PS were analyzed by repeated-measures ANOVA, *p* < 0.05.

**Table 6:** Correlation between metabolic and ventilatory parameters.

	EE (kcal)	VO_2_ (ml/min)	VCO_2_ (ml/min)
P0.1 (cmH_2_O)			
*r*	0.442^*∗*^	0.424^*∗*^	0.457^*∗*^
*p*	<0.001	<0.001	<0.001

EE, energy expenditure; VO_2_, oxygen consumption; Vt, tidal volume; RR, respiratory rate; P0.1, airway occlusion pressure at 0.1 sec. ^*∗*^*p* < 0.05.

## Data Availability

The data that have been used are confidential currently and may be ready to share later.
